# MicroRNA regulation of progesterone receptor in breast cancer

**DOI:** 10.18632/oncotarget.15657

**Published:** 2017-02-23

**Authors:** Avital Gilam, Ayelet Shai, Itamar Ashkenazi, Liat Appel Sarid, Assi Drobot, Amitai Bickel, Noam Shomron

**Affiliations:** ^1^ Sackler Faculty of Medicine, Tel Aviv University, Tel Aviv, Israel; ^2^ Oncology Department, Galilee Medical Center, Nahariya, Israel; ^3^ Faculty of Medicine, Bar Illan University, Zefad, Israel; ^4^ Hillel Yaffe Medical Center, Hedera, Israel

**Keywords:** breast cancer, progesterone receptor, microRNA, miR-181a, miR-23a

## Abstract

Hormone receptor status is of significant value when deciding on anti-estrogenic adjuvant therapy for breast cancer tumors. However, while estrogen receptor (ER) regulation was intensively studied, the regulation of progesterone receptor (PR) levels has not been extensively investigated. MicroRNAs (miRNAs, miRs) are post-transcriptional negative regulators of gene expression involved in diverse cellular processes. The aim of this study was to identify miRNAs that regulate PR in breast cancer.

We mapped potential miRNA binding sites for miR-181a, miR-23a and miR-26b on PR mRNA and demonstrated a direct regulation of PR by these three miRNAs by *in-vitro* Luciferase binding assays. Over-expression of each miRNA in MCF-7 cells resulted in a reduction in the expression levels of PR mRNA. Then, expression levels of these miRNAs were measured in Formalin-Fixed, Paraffin-Embedded (FFPE) samples of 29 ER-positive breast cancer tumors and adjacent normal breast tissues. A significant reciprocal correlation between PR mRNA and the miRNA levels were identified suggesting a role for miR-181a, miR-23a and miR-26b in PR regulation in breast cancer. Moreover, the average expression fold-changes of the three miRNAs between cancerous and normal tissues displayed an opposite trend when analyzing according to Immuno-histochemistry(IHC) status. Furthermore, miR-181a and miR-26b were found to be over-expressed in most tumor tissues supporting their role in ER-positive breast cancer development. We conclude that miR-181a, miR-23a and miR-26b act as negative regulators of PR expression in ER-positive breast cancer. The diagnostic and prognostic potential of these miRNAs in breast cancer should be further evaluated.

## INTRODUCTION

Breast cancer is the most frequent cancer in women, with more than 200,000 cases diagnosed per year in the USA. Hormone receptor status is of paramount importance when deciding on breast cancer treatment. The estrogen receptor (ER) status has long been recognized as an important factor in prognosis and management of breast cancer and breast tumors that express the ER are often treated with anti-estrogenic drugs in the adjuvant and metastatic setting. During the last four decades studies have tested the importance of progesterone receptor (PR) status in the decision making regarding the therapy for breast cancer patients [1, 2]. In most studies, PR nuclear staining correlated with the likelihood of benefit from anti-estrogenic therapies [2–5]. In addition, exposure to progesterone is a well-recognized risk factor for postmenopausal breast cancer [6].

The PR status is specifically important for distinguishing between breast tumor subgroups that might benefit differently from adjuvant anti-estrogenic therapies: the ER+/PR- subgroup was recognized as less responsive to endocrine therapy (particularly tamoxifen) than ER+/PR+ subgroup [5, 7–9], and it has been shown that the strength of nuclear ER and PR staining is related to the likelihood of benefit from these drugs [10].

In the past, biochemical assays were used to detect PR expression in breast tumors. However, each assay used different parameters for defining “positive” or “negative” expression of PR. In the last two decades, the common method used for ER/PR status determination is Immuno-histochemistry (IHC), however there is no gold standard for assessing PR IHC status, and diverse methods and cut-off points for defining PR status as positive or negative are being employed in a non-uniform manner in the clinical setting [11].

The regulation of PR expression from the *PGR* gene is poorly understood. In breast cancer, expression of PR is thought to be governed by the estrogen receptor, and PR expression serves as an indicator for an intact estrogen-ER signaling pathway [12]. Signaling by growth factor receptors such as the EGFR family and IGF-1R and crosstalk between ER and these signaling pathways were shown to down-regulate PR [13]. An interaction between the mTOR pathway, an established mediator of hormone resistance in breast cancer, and PR has also been suggested [14]. However, the intracellular mechanisms that result in low PR expression in response to these signaling events are incompletely defined.

MicroRNAs (miRNAs, miRs) are small, naturally occurring, non-coding molecules, about 22 nucleotides long, which negatively regulate gene expression. They exert their functional role by binding their ‘seed’ region (nucleotides 2-7 of the miR) to short conserved complementary sequences in 3’ untranslated regions (UTRs) of downstream target mRNAs, which follows by translation inhibition or mRNA degradation. These short RNAs have the potential to target hundreds of genes and thus they are under tight and dynamic regulation [15]. In recent years it was shown that many malignant tumors, including breast cancers, have disrupted miRNA regulation [16–18]. Moreover, miRNA expression profiles enable successful classification of poorly differentiated tumors, whereas mRNA profiles were highly inaccurate [19]. These findings highlight the potential of miRNA profiling as cancer diagnosis and prognosis marker as well as therapeutic target [20].

Studies suggest that miRNAs expression profile changes with fluctuating steroid hormone levels, in normal steroid responsive tissues such as the endometrium and the mammary gland [21–24]. MiRNAs were shown to be involved specifically in the function of the progesterone receptor during physiologic and pathologic conditions [25]. In addition, experiments suggest that miRNAs mediate the carcinogenic effects of progesterone in the breast [26, 27] and it was shown that there are clear differences in miRNA levels in ER+/PR+ breast tumors *versus* triple negative breast tumors [28, 29]. Furthermore, several studies [17, 28, 30] demonstrated a correlation between the levels of specific miRNAs and PR status.

Recent experiments generated a list of miRNAs that can be regulated by progesterone and thereby mediate the cellular effect of the progesterone-PR pathway *via* targeting a wide variety of target genes. This list included some miRNAs which are also regulated by the PR itself (reviewed by D.R Cochrane et al. 2012 [26, 31]). However, little is known regarding miRNAs that target the PR and how they affect PR levels and function. Thus far, few studies looked at the regulation of PR expression by miRNAs. Maillot et al. suggested that miR-181a and miR-26a are negative regulators of PR expression in breast cancer cell lines [32]. Others suggested that miR-181a, miR-26a, miR-513-5p and miR-126-3p regulate PR expression in endometrial carcinogenesis and during mammary development [26, 32–34].

In this study, we sought to identify miRNAs that regulate PR expression, and to elucidate their role in ER positive breast cancer. Our *in-vitro* studies point at a direct regulation of PR by miR-181a, miR-23a and miR-26b, and show their effect on PR levels in cell lines. Further investigations using several analyses of miRNAs and PR expression in breast cancer samples also propose a functional regulation of PR by the three miRNAs during breast cancer development, and support a common regulation of these three miRNAs’ expression in breast tissue.

## RESULTS

The *PGR* gene has a very long (9492nt) 3’-untranslated region (3'UTR) with only four conserved miRNA binding sites for four miRNA families [32]: miR-181, miR-26, miR-23, and miR-135 (also identified by online target site predictors such as TargetScan 5.2, miRWalk and miRanda). In this study we focused on miR-181a, miR-23a and miR-26b, and tested their relevance to the regulation of PR in breast cancer.

**Figure 1:**
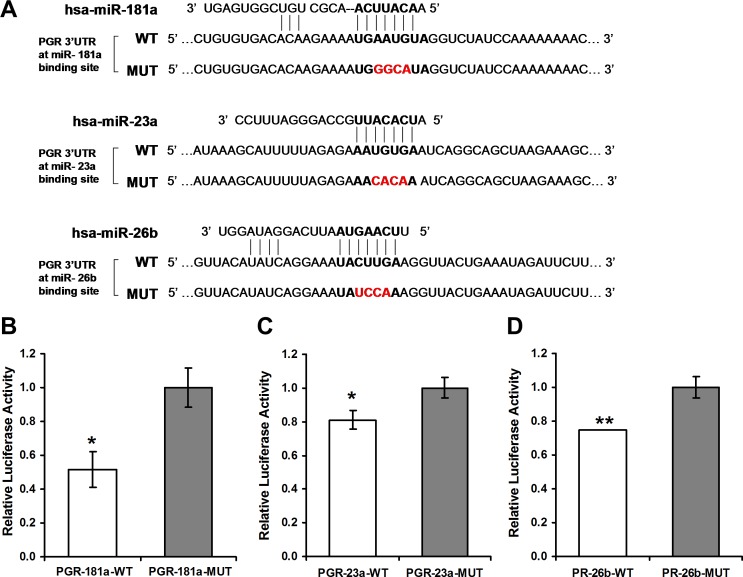
Luciferase reporter assays **A**. Sequences of Ranilla/Firefly Luciferase psiCHECK2 constructs under regulation of *PGR* 3'UTRs that were used for transient reporter assay experiments. WT and Mutant (Target-deletion) alleles for each microRNA binding site are presented. **B**. Luciferase activity 48 hours following co-transfection with miRNA-181a in combination with either of the *PGR* 3'UTR constructs in HeLa cells. **C**., **D**. Luciferase activity 24 hours following co-transfection with miR-23a or miR-26b, respectively, in combination with the indicated *PGR* 3'UTR constructs in MCF-7 cells. Values are presented as mean ± SEM, *n* = 3, *p < 0.05, **p < 0.01, two-tailed student's *t*-test.

Initially, luciferase reporter assay was performed to assess if miR-181a, miR-23a and miR-26b exert a direct functional regulation on PR expression. Regions of miRNA binding sites on the 3'UTRs of the *PGR* gene were cloned into the Ranilla/Firefly Luciferase psiCHECK2 construct. Then, four nucleotides of the miRNA binding site on the 3'UTR were swapped in order to abrogate the binding. These mutant constructs were used as negative controls in the assay (see constructs and sequences in Figure 1A). The constructs were co-transfected with constructs containing the genes of the relevant miRNA into MCF-7 or HeLa cells. A significant decline in luciferase activity following co-transfection of the wild type (WT) constructs and the miRNA genes compared with co-transfection of the mutated constructs was noted, albeit at a different time frame for each miRNA (Figure 1B-1D). A reduction in luciferase activity was noted 24 hours and 48 hours following co-transfection with miR-181a (Figure 1B). Following co-transfection of miR-23a and miR-26b a reduction in luciferase activity was noted only after 24 hours (Figure 1C and 1D).

**Figure 2:**
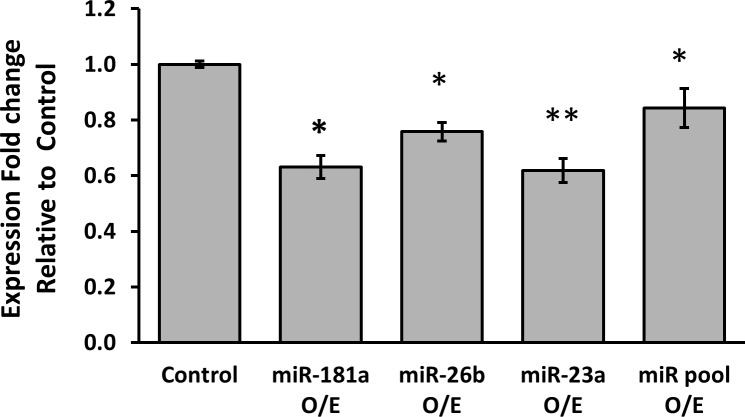
Average expression fold change of PR mRNA, 6 hours following over-expression of each miRNA MCF-7 cells were transfected by miR-vec miR-181a, miR-vec miR-26b, miR-23a mimic or miR-vec miR-181a/miR-26b/miR-23a pool, as well as by Empty miR-vec plasmid or Scrambled sequence as control. Levels of PR mRNA were determined by quantitative RT-PCR analysis. The comparative threshold cycle (Ct) method was used for quantification. Values are presented as mean±SEM, n≥5, *p < 0.05, **p < 0.01, two-tailed student's *t*-test.

Next, the effect of over-expression of the different miRNAs on PR mRNA levels was tested. MCF-7 cells were transfected with either miR-181a, miR-26b, miR-23a or with all the three miRNAs together and PR mRNA expression levels were determined by quantitative RT-PCR. PR mRNA levels were significantly reduced six hours following transfection of each of the miRNAs (Figure 2). While the effect of over-expression of miR-181a on PR expression lasted 12 hours, levels of the PR mRNA returned to baseline 12 hours after transfection of miR-26b and miR-23a. PR levels returned to baseline 24 hours following transfection of each of the miRNAs. Cotransfection of all 3 miRNAs did not result in an additive or synergistic effect on PR expression.

Taken together, these results demonstrate a relatively rapid regulation of PR expression by miR-181a, miR-23a and miR-26b. This is consistent with the results of the luciferase assay described above, and suggests that miR-181a has a longer lasting effect on PR expression compared with miR-23a and miR-26b.

In order to test if PR is regulated by miRNAs *in-vivo*, we analyzed expression levels of PR mRNA, miR-181a, miR-23a and miR-26b in samples from 29 ER positive, HER2 negative breast cancer patients (see Table 1). For each patient, we collected two FFPE samples: one from breast carcinoma tissue and one from adjacent normal breast tissue. In total, 29 tumor tissues, 24 adjacent normal breast tissues and 20 pairs of tumor and normal samples of the same patient were included in the results presented below. Samples were excluded from the analysis due to technical reasons, mainly lack of RNA. Outlier samples were defined as samples that deviated from average by more than 1.5 standard deviations, and were remove from downstream analyses.

**Table 1:** Clinical characteristics of breast cancer patients

Patient no.	Age at Diagnosis (years)	Histological subtype	Grade	Stage	Tumor size (cm)	ER status	PR status	HER2 status	Metastasis node count	Menopause	Follow up	Recurrence
1	61.5	IDC	2	I	0.7	positive	negative	negative	0	yes	4.2 years	No
2	69.9	IDC	2	I	2	positive	negative	negative	0	yes	4 years	No
3	69.5	IDC	2	I	1	positive	negative	negative	0	yes	3.5 years	No
4	65.3	ILC	1	IIA	3	positive	negative	negative	0	yes	3 years	No
5	62.1	IDC	3	IIA	2.8	positive	negative	negative	0	yes	5.5 years	No
6	61	IDC	2	I	1.8	positive	negative	negative	0	yes	2 years	No
7	59.4	IDC	2	IIA	1.7	positive	negative	negative	0	yes	5.5 years	No
8	50.7	IDC	1	I	1.7	positive	positive	negative	0	yes	4.5 years	No
9	57	IDC	3	1	1.8	positive	positive	negative	0	yes	3.8 years	No
10	34.5	IDC	2	IIB	3	positive	positive	negative	1	no	4 years	No
11	52.6	IDC	2	IIA	2	positive	positive	negative	0	no	4.5 years	No
12	73	IDC	2	1	1.2	positive	negative	negative	0	yes	5.5 years	No
13	60.2	IDC	1	I	1	positive	positive	negative	0	yes	2 years	No
14	83.7	IDC	1	I	0.9	positive	negative	negative	0	yes	2.5 years	No
15	75	IDC	1	I	1.8	positive	negative	negative	0	yes	1.1 years	No
16	55.5	IDC	3	IIA	3.5	positive	negative	negative	0	yes	0.9 years	No
17	61	IDC	2	IIA	2.2	positive	positive	negative	0	yes	0.5 years	No
18	71.9	IDC	3	IIB	5	positive	positive	negative	0	yes	0.4 years	No
19	85	IDC	2	IIB	3	positive	positive	negative	1	yes	0.4 years	No
20	69.5	IDC	3	IIA	2	positive	positive	negative	0	yes	0.4 years	No
21	79.7	IDC	3	IIB	3	positive	negative	negative	1	yes	0.5 years	No
22	69	IDC	1	IA	1.3	positive	positive	negative	0	yes	1.5 years	No
23	48.5	IDC	2	IIA	1.6	positive	positive	negative	2	no	1.2 years	No
24	57	IDC	1	IA	1.7	positive	positive	negative	0	yes	1.4 years	No
25	37.8	IDC	3	IIIA	8	positive	positive	negative	12	no	2 years	yes
26	76.5	ILC	1	IIA	3.1	positive	positive	negative	0	yes	1 year	No
27	47.5	IDC	2	IIA	1.6	positive	positive	negative	2	no	1.2 years	No
28	55	IDC	2	IIB	3	positive	negative	negative	2	yes	1.2 years	No
29	88	IDC	3	unknown	1	positive	negative	negative	unknown	yes	1 year	No

Abbreviations: IDC, Invasive ductal carcinoma; ILC, Invasive lobular carcinoma; ER, Estrogen receptor; PR, progesterone receptor; HER2, human epidermal growth factor receptor 2.

**Figure 3:**
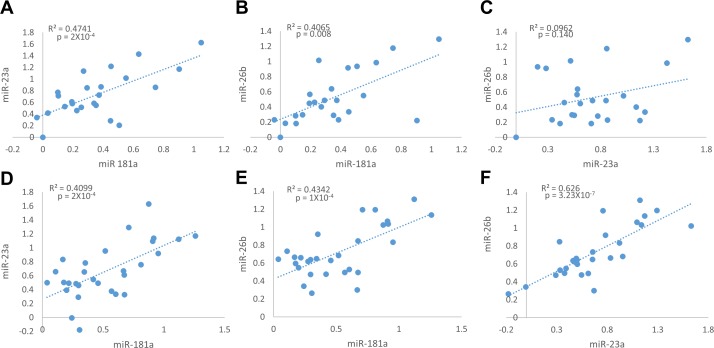
Positive correlation between Relative expression of miR-181a, miR-23a and miR-26b in adjacent normal tissue samples (A.-C., *n* = 24), and in breast cancer tumor samples (D.-F., *n* = 29) Levels of miRs and PR mRNA were determined by quantitative RT-PCR analysis. The comparative threshold cycle (Ct) method was used for quantification. Values are presented in log scale. Lines represent the linear regression analysis; R^2^ (Goodness-of-fit) for the linear regression analysis and p-values are indicated.

We first tested the relative expression of miR-181a, miR-23a and miR-26b in tumors and normal breast tissues. MiR-181a relative expression was positively and significantly correlated with the relative expression of miR-23a and miR-26b in the normal breast tissues (Figure 3A and 3B), and in the tumor tissues (Figure 3D and 3E). No correlation was observed between miR-23a and miR-26b in the normal samples (Figure 3C), but they strongly and highly significantly correlated in the tumor samples (Figure 3F). This suggests that the expression of these three miRNAs shares a common regulatory pathway, specifically during cancer development. Surprisingly, no correlation was observed between the relative expression of each of the miRNAs and PR mRNA.

We noted that the expression of the PR mRNA was highly variable between patients in the normal breast tissues (Supplementary Figure S1). We thus decided to calculate the expression fold change of PR mRNA as well as of miR-181a, miR-23a and miR-26b in each patient's breast cancer tumor sample relative to its adjacent normal tissue [35] (for fold change values see Supplementary Table S1) and to use this to further analyze the interplay between the expression of the three miRNAs and PR.

**Figure 4:**
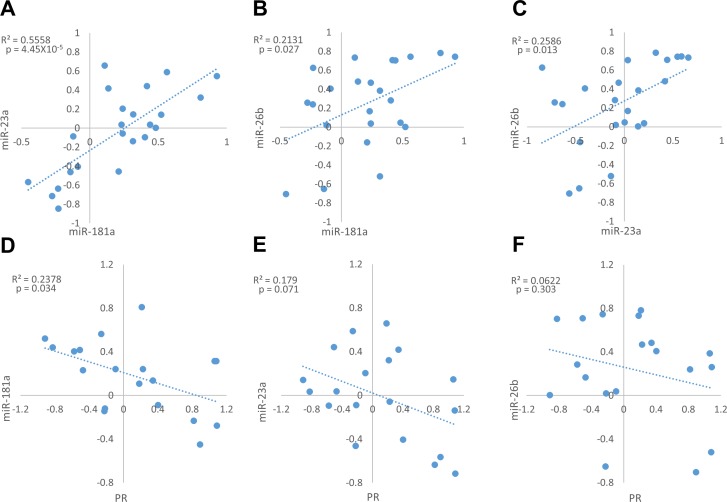
Expression fold change of miR-181a, miR-23a, miR-26b and PR mRNA in each breast cancer tumor sample and its’ adjacent normal tissue **A.-C**. Correlation between miRs expression fold change (*n* = 23). **D**.-**F**. Correlation between each miR and PR mRNA expression fold change (*n* = 20). Levels of miRs and PR mRNA were determined by quantitative RT-PCR analysis. The comparative threshold cycle (Ct) method was used for quantification. Values are presented in log scale. Lines represent the linear regression analysis; R^2^ (Goodness-of-fit) for the linear regression analysis and p-values are indicated.

A positive and significant correlation was observed between the expression fold changes (tumor *vs*. adjacent normal tissue) of all tested miRNAs (Figure 4A-4C), with the strongest and highest significant correlation between miR-181a and miR-23a (Figure 4A).

Notably, miR-181a expression fold changes were negatively and significantly correlated with PR mRNA fold changes between normal breast and breast cancer tissues (Figure 4D), suggesting that miR-181a down-regulates the expression of PR in breast cancer *in-vivo*. However, weak and insignificant negative correlations were observed between miR-23a or miR-26b expression fold changes and PR mRNA expression fold changes (Figure 4E-4F).

**Figure 5:**
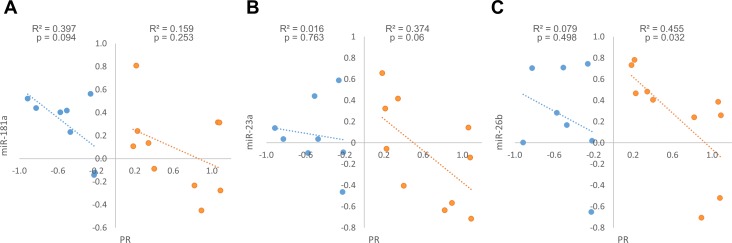
Expression fold change of miR-181a, miR-23a, miR-26b and PR mRNA in each breast cancer tumor sample and its’ adjacent normal tissue, in PR mRNA up-regulation samples A.-C. (*n* = 10) and in PR mRNA down-regulation samples D.-F. (*n* = 8) Levels of miRs and PR mRNA were determined by quantitative RT-PCR analysis. The comparative threshold cycle (Ct) method was used for quantification. Values are presented in log scale. Lines represent the linear regression analysis; R^2^ (Goodness-of-fit) for the linear regression analysis and p-values are indicated.

When we examined the expression fold changes of PR in tumors compared to their adjacent normal breast tissue, two distinct patterns emerged - tumors in which PR mRNA expression is higher compared to the normal breast tissue (up-regulated) and tumors in which it is lower (down-regulated). PR up-regulated tumors were defined as tumors in which PR mRNA expression fold change was above 1.2 and PR down-regulated tumors were defined as tumors in which PR mRNA expression fold change was below 0.8 (for fold change values see Supplementary Table S1). We next looked at the correlation between the expression fold changes of the miRNAs and PR mRNA separately in tumors that displayed PR up-regulation and those that displayed PR down-regulation. Interestingly, we found opposite trends in these two breast tumor subgroups: while PR up-regulated tumors displayed a significant negative correlation between miR-26b and PR mRNA expression fold changes (Figure 5C), a marginally significant negative correlation between miR-23a and PR mRNA expression fold changes (Figure 5B), and weak and insignificant negative correlation between miR-181a and PR mRNA expression fold changes (Figure 5A), PR expression fold changes correlated negatively only with miR-181a expression fold changes (marginally significant) in PR down-regulated tumors (Figure 5A). This suggests a stronger regulation of PR by miR-23a and miR-26b in tumors in which PR is up-regulated and an alternative regulation by miR-181a in tumors in which PR is down-regulated.

**Figure 6:**
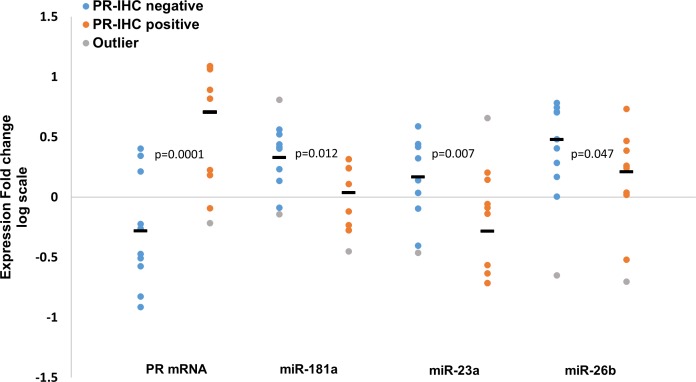
Expression fold change (tumor *vs*. adjacent normal tissue, log scale) of PR mRNA, miR-181a, miR-23a and miR-26b in PR IHC-negative (n = 10) and PR IHC-positive (n = 10) breast cancer patients Line “—“ represents the average of each group. Levels of miRs and PR mRNA were determined by quantitative RT-PCR analysis. The comparative threshold cycle (Ct) method was used for quantification. *P*-values by two-tailed student's *t*-test are indicated.

We further explored the expression values according to PR expression by IHC. Although the compatibility between the measured mRNA expression fold changes (tumor *vs*. adjacent normal tissue) and the PR IHC status is high (70% of the PR up-regulated tumors were defined as PR IHC-positive, and 75% of the PR down-regulated tumors were defined as PR IHC-negative, see Supplementary Table 1), not all samples follow this pattern. Therefore, we compared the expression fold changes of PR mRNA, miR-181a, miR-23a and miR-26b in the PR IHC-positive and negative groups. As expected, PR mRNA average expression fold change was significantly higher in the PR IHC-positive group compared to the PR IHC-negative group (Figure 6). Importantly, an opposite trend was observed for all three miRNAs where their average fold-changes were significantly lower in the PR IHC-positive group compared to the PR IHC-negative group (Figure 6). This opposite trend for all three miRNAs and PR mRNA again points to a common role for miR-181a, miR-23a and miR-26b in PR regulation.

Notably, none of the miRNA levels were reduced in tumors of the PR IHC-negative group (Supplementary Table 2), implicating a potential function of all three miRNAs in regulating PR levels in this tumor subgroup. However, consistent with our previous results, the expression fold changes of PR mRNA negatively and significantly correlated only with those of miR-181a when analyzing the PR IHC-negative samples (Supplementary Figure S2). This further supports the notion that miR-181a is a negative regulator of PR in breast cancer tumors.

**Figure 7:**
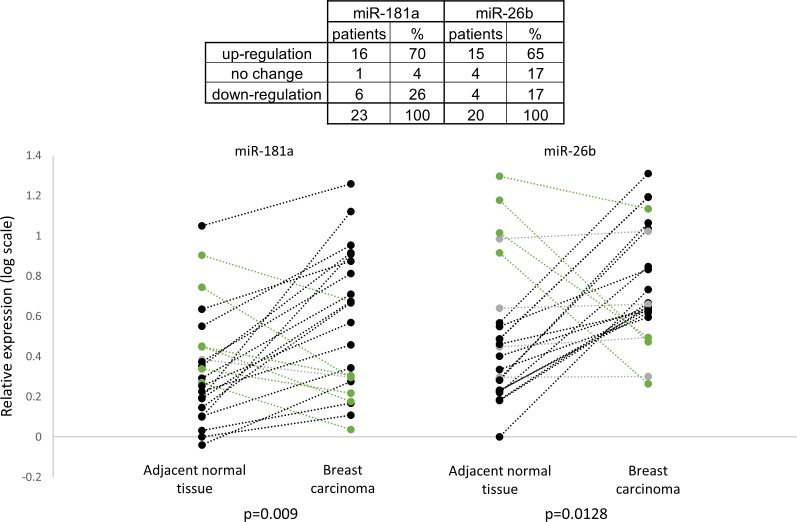
Relative expression of miR-181a and miR-26b in breast carcinoma samples and in adjacent normal tissue samples (*n* = 23) MiR-181a and miR-26b abundances for each paired adjacent normal tissue and tumor tissue are shown separately in the left and the right parts and connected by a dash line. Levels of miR-181a and miR-26b were determined by quantitative RT-PCR analysis. The comparative threshold cycle (Ct) method was used for quantification. Values are presented in log scale. P-values by two-tailed paired student's *t*-test are indicated.

We further analyzed the expression pattern of the miRNAs in adjacent normal tissue samples and in breast carcinoma samples. Figure 7 shows that the relative expression of miR-181a and miR-26b was higher in breast cancer samples compared to adjacent normal tissue samples in most of the patients (16 and 15 patients out of 23, respectively). Overall, the relative expressions of miR-181a and miR-26b in breast carcinoma samples were significantly higher than in the adjacent normal tissue samples (Figure 7). These results implicate a potential role for miR-181a and miR-26b in breast cancer development.

## DISCUSSION

The regulation of the progesterone receptor in breast cancer has not been thoroughly studied. Recently, a small number of studies [26, 32, 34] addressing PR regulation by miRNAs in breast cancer were published. However, most studies were correlative and did not directly demonstrate PR as a target for a specific microRNA [17, 28, 30, 36]. Our study is one of the first to address this issue by testing the *in-vitro* influence of specific miRNAs on PR expression, and the *in-vivo* correlation between miRNAs and PR expression in human breast cancer samples.

We focused on three miRNAs, miR-181a, miR-23a and miR-26b. These three microRNAs have one conserved binding site on the 3'UTR of the *PGR* gene, and have been suggested to have a role in breast cancer biology: miR-181 was found to be involved *in-vitro* in tamoxifen resistance [37], to effect metastatic potential [38, 39], and to repress the DNA damage response in aggressive breast cancer [40]; miR-23 was found to be more than five-fold over-expressed in breast cancer sample compared to adjacent normal tissue [41]; and, blockade of miR-26 with antisense inhibitors leads to an increased apoptotic response while an excess of miR-26 decreases the apoptotic response in MCF-7 breast cancer cell line [42], suggesting an impact of this miRNA on tumor formation.

Our *in-vitro* studies strongly support the hypothesis that the three miRNAs tested regulate PR expression. We showed by luciferase reporter assay that the presence of each of the miRNAs leads to a decrease in luciferase signal, and that the miRNA binding sites are essential for PR regulation. This effect was significant for all three miRNAs (miR-181a, miR-23a and miR-26b), with a significant decrease of 20-50% in luciferase expression. In addition, the over-expression of each of the miRNAs separately in MCF-7 cells leads to a significant decrease in PR mRNA levels. The fact that over-expression of all the miRNAs simultaneously has a comparable effect to that of individual miRNA over-expression indicates an overlap in function.

Previous results suggested a role for miR-181a in PR expression regulation. miR-181a was shown to directly regulate PR expression by luciferase reporter assay in HEK-293T cells [32], and in Ishikawa cells [33]. Over-expression of miR-181a repressed PR mRNA and protein expression in MCF-7 cells [32] and in Ishikawa cells [33], while over-expressing anti-miR-181a led to an increase in PR expression in MCF-7 cells [32]. Our results validate *PGR* as a direct target of miR-181a and support these previous results.

Our study is the first to demonstrate a direct regulation of *PGR* by miR-23a or miR-26b. Notably, previous evidence demonstrated a regulation of PR expression by miR-26a, a family member of miR-26b [32].

Our *in-vivo* studies exploited samples from patients with ER-positive breast cancer diagnosed in stages I-III. We analyzed the relative expression as well as the expression fold change of PR mRNA and the three miRNAs in breast tumor tissues and in normal tissues (the tissue surrounding the tumors).

The positive and significant correlations observed between the expression fold changes of all the three miRNAs suggest that their expression shares a common regulatory pathway. Moreover, the strong and significant positive correlation between the relative expressions of miR-181a and miR-23a or miR-26b in the normal surrounding tissues, and the fact that the relative expressions’ correlation between all the three miRNAs is stronger and highly significant when comparing the tumor samples, suggest that they are jointly regulated in normal breast tissues, and to an even higher extent in malignant breast tissue.

Each of these miRNAs is located at different genomic loci: hsa-miR-181a is located on chromosome 1 and on chromosome 9; miR-23a is located on chromosome 19; and, miR-26b is located on chromosome 2. This means that these miRNAs are not expressed from a single cluster and are not regulated by one common promoter. However, it seems that miR-181a and miR-26b share conserved transcription factor binding sites upstream of their gene for transcription factors FOXQ1, FOXJ2 and Tal-1. These are located at < 5kb upstream of the miRNA genes.

Two other studies suggested a common regulation of these miRNAs in the context of breast cancer. Maillot et al, 2009 [32], reported that the expression of the three tested miRNAs, miR-181a, miR-23a and miR-26b, as well as their family members miR-181b, miR-23b and miR-26a, respectively, are regulated by estrogen. These miRNAs were down-regulated following 17β-estradiol (E_2_) treatment in ER-positive breast cancer cell lines (MCF-7, T47D, ZR-75-1 and BT-474) and at least one ERα binding site is located at < 50kb around these miRNAs sequences [43]. In addition, these miRNAs, among others, were found to be up-regulated in breast cancer cell lines following exposure to conditions of hypoxia, a well-known tumor microenvironment factor [42]. All three miRNAs have at least one (and up to 10 for miR-26b) hypoxia response elements (HRE) upstream of the miRNA genes ( < 5kb).

When we analyzed PR expression in normal breast tissue we observed a marked variability between patients. When we compared PR expression in tumors and their adjacent normal breast tissue, two distinct patterns emerged - tumors in which PR is up-regulated and tumors in which PR is down-regulated compared to the normal breast. This intriguing and novel finding suggests that understanding the differences between women in the response of normal breast to hormonal stimuli is important in order to understand breast cancer biology.

Since miRNAs are negative regulators of gene expression, a negative correlation between PR mRNA expression and miR-181a, miR-23a or miR-26b expression in breast tumor tissues would suggest a role for these miRNA in breast cancer *in-vivo*. Such a correlation was observed in the expression fold changes between PR mRNA and miR-181a when analyzing the whole cohort. This result strongly supports a role for miR-181a in PR expression regulation *in-vivo* during breast cancer transformation. Further analysis examining PR up-regulated and PR down-regulated tumors separately showed that these two tumor subgroups displayed opposite trends in the expression fold changes of miR-181a, miR-23a and miR-26b: PR mRNA was correlated with miR-26b and miR-23a in PR up-regulated tumors, and with miR-181a in PR down-regulated tumors. These results suggest that tumors exploit overlap mechanisms for PR regulation depending on the relation of PR expression between tumor and normal breast tissue.

MiRNAs can regulate gene expression by inhibiting protein translation. Therefore, we analyzed the relationship between the miRNAs and PR according to the tumors’ PR IHC-status, which reflects PR protein expression. Our analysis shows that the averages of the expression fold changes of all three miRNAs were significantly lower in the PR IHC-positive group compared to the PR IHC-negative group. This further supports a role for miR-181a, miR-23a and miR-26b in PR regulation in breast cancer.

The levels of miR-181a and mir-26b are higher in almost all samples compared to the adjacent normal breast tissue, supporting a role for miR-181a and miR-26b in ER-positive breast cancer development. This finding is consistent with the anti-apoptotic effect of miR-26b in MCF-7 ER-positive breast cancer cell line [42]. Two previous studies found that miR-26b is under-expressed in human breast cancer compared to normal tissue surrounding the tumors, and suggested that miR-26b induces apoptosis [44] and inhibit proliferation [45] in breast cancer cell lines. However, these studies were performed using specimens from patients with diverse breast cancer histological sub-types and ER/PR/HER2 receptor status [44], while our work focused on a subgroup of ER-positive, HER2 negative breast cancer patients. An additional study showed that the average of miR-26b expression levels is lower in breast cancer tissue specimens compared to normal tissues, and suggested that miR-26b inhibit breast cancer cell growth [46].

Taken together, our work presents accumulating evidence for miR-181a, miR-23a and miR-26b as negative regulators of PR expression in ER-positive breast cancer development. As mentioned, PR expression is regulated by several factors, including the signaling pathways of ER, EGFR, IGF-1R and others. All these pathways might alter PR levels in the tumor. We propose that miR-181a, miR-23a and miR-26b take part in the mechanism that reduces PR expression. Additionally, our findings support a common regulation of the expression of these three miRNAs in normal breast tissue, and to an even higher extent in cancerous breast tissue. Further analysis on a larger cohort of breast cancer patients is necessary to assess the diagnostic and prognostic potential of miR-181a, miR-23a and miR-26b in breast cancer.

## MATERIALS AND METHODS

### Patients and tissue samples

Samples and clinical data were obtained from Galilee Medical Center, Nahariya, Israel and Carmel Medical Centers, Haifa, Israel. Overall, 29 women that were diagnosed with stage I-III, ER positive, HER2 negative breast cancer were included in this study. Median age at breast cancer diagnosis was 63.4 ± 13.2 years (mean + SD) (range 34.5-88 years). Patients that received neoadjuvant chemotherapy were excluded. The pathological specimen was reviewed by a pathologist. For each patient, two Formalin-Fixed, Paraffin-Embedded (FFPE) tissue blocks were selected: one containing only tumor tissue and one containing only normal breast tissue. Twenty 5uM wide slices of FFPE tissue were dissected from each block. All studies were approved by institutional ethical committees.

### Immuno-histochemistry

PR status was determined using standard Immuno-histochemistry. The Pathology laboratories in participating centers undergo yearly external quality assessments under the College of American Pathologists (CAP) program. Tumors were defined as PR negative when no PR expression was observed by IHC.

### RNA extraction and qRT-PCR

RNA was extracted from Formalin-Fixed, Paraffin-Embedded (FFPE) tissues using the RecoverAll Total Nucleic Acid Isolation Kit (Applied Biosystems, ABI) and from cell-lines using Trizol (Bio-Lab). The final RNA concentration and purity were measured using a NanoDrop ND-1000 spectrophotometer (NanoDrop Technologies, Thermo Scientific).

Reverse transcription of mRNA was done using random-primer and SuperScript III reverse transcriptase (Invitrogen). For specific mature miRNAs reverse transcription was carried out using TaqMan miRNA Assays according to manufacturer's protocol (Applied Biosystems; ABI). Single miRNAs/mRNAs expression were tested similarly using TaqMan Universal PCR Master Mix (No AmpErase UNG; Applied Biosystems) or SYBR green PCR master mix (Applied Biosystems), respectively. The PCR amplification and reading was done using the Step-One thermal cycler (Applied Biosystems). Specific primers for mRNA expression detection were ordered from Sigma: PR Forward primer - TCAGTGGGCAGATGCTGTATTT; PR reverse primer - GCCACATGGTAAGGCATAATGA. Expression values were calculated based on the comparative threshold cycle (Ct) method [47]. MiRNAs levels were normalized to U6 expression levels.

### Cloning and site-directed mutagenesis

Three regions (∼500bps each) flanking three predicted binding site for the miRNAs hsa-miR-181a-5p, hsa-miR-23a-3p and hsa-miR-26b-5p on the 3'UTR of *PGR* gene were PCR-amplified from genomic DNA of MCF-7 human breast cancer cell line using Phusion High-Fidelity DNA Polymerase (Finnzymes). These DNA fragments were fused downstream to the Renilla gene of the psiCHECK2 vector (Promega). In each *PGR* 3‘UTR psiCHECK2 vector, four nucleotides were mutated using the QuickChange Lightning Site-Directed Mutagenesis kit (Agilent) in the seed region of the miRNA binding site. The vectors were then sequenced to verify that the correct sequence is present in all plasmids (see Figure 1).

### Cell cultures and transfections

HeLa cells (human cervical carcinoma cells) and MCF-7 (human breast cancer cell-line) were grown in DMEM medium supplemented with 10% FBS and antibiotic (Streptomycin 100mg/ml, Penicillin 100mg/ml) (Biological Industries). HeLa cells were obtained through the NIH AIDS Reagent Program, Division of AIDS, NIAID (#3522). MCF-7 cell line was kindly gifted from Prof. Ilan tsarfaty, Sackler Faculty of Medicine, Tel Aviv University, Israel.

For luciferase assay experiments, cells were plated in 24-wells plates at a concentration of 0.5×10^5^ cells/well. Transfection was done using Lipofectamine 2000 (Invitrogen), 5ng of the psiCHECK2 relevant clone, 10ng of pEGFP and 485ng of miR-Vec-181a-5p, miR-Vec-23a-3p or miR-Vec-26b-5p. The miR-Vec retroviral vector contains the genomic region of the pri-miRNA under a strong CMV promoter (provided by Dr Reuven Agami, The Netherlands Cancer Institute). Transfection efficiencies were measured by co-transfecting a GFP expressing plasmid. Transfection efficiency was at a minimum of 70% for experiments to proceed. Twenty-four or forty-eight hours following transfection, cells were taken for Luciferase activity determination.

For over-expression experiments, cells were plated in 12-wells plates at a concentration of 1×10^5^ cells/well. Transfection was carried-out using Lipofectamine 2000 (Invitrogen), 1ug miR-Vec vector (miR-Vec-181a-5p, miR-Vec-26b-5p, miR-Vec-23a or an empty non-miRNA harboring miR-Vec as control) or 60pmole synthetic sequences (hsa-miR-23a-3p or scrambled sequence as control) (Ambion). Cells were isolated for RNA extraction 6, 12 and 24 hours following transfection.

### Luciferase reporter assay

Luciferase assay was performed in 96-wells plates 24-48h following transfection using the Dual Luciferase Reporter Assay kit (Promega) and following the manufacturer protocols. The LUMIstar Omega Luminometer (BMG LabTech) was used to read the intensities. The Renilla luciferase results were normalized to the values of the Firefly luciferase.

### Data analysis

Means and standard deviation values for each experiment were calculated by MS Excel (MS Office 2003). Goodness-of-fit (R^2^) and p-values for each linear regression analysis (two-tailed Pearson's linear correlation) were calculated by SPSS statistical software (Version 15).

## SUPPLEMENTARY MATERIALS FIGURES AND TABLES


