# Supplementary material for: MicroRNA regulation of progesterone receptor in breast cancer
Source: Oncotarget. 2017 Feb 23;8(16):25963–76. doi: 10.18632/oncotarget.15657 (PMC5432230; doi:10.18632/oncotarget.15657)
Supplement: Supplementary file 1 [file oncotarget-08-25963-s001.pdf]

MicroRNA regulation of progesterone receptor in breast cancer

Supplementary Material

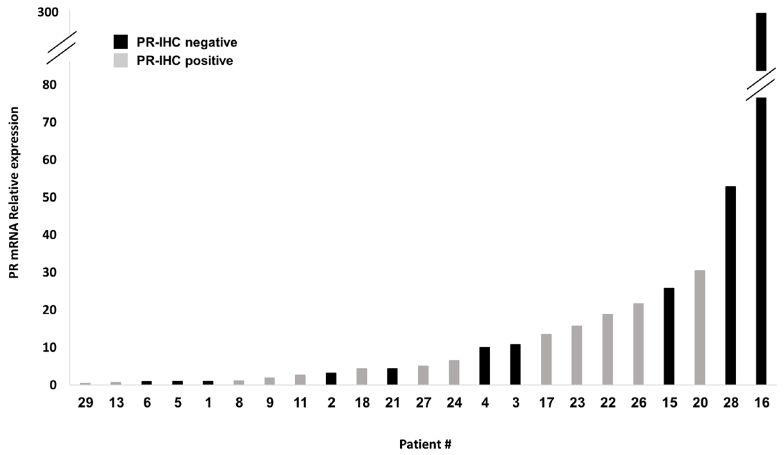

Supplementary Figure S1: Expression levels in adjacent normal tissue samples. Relative expression of PR mRNA in PR IHC-negative and positive groups in individual patients. Levels of PR mRNA were determined by quantitative RT-PCR analysis. The comparative threshold cycle (Ct) method was used for quantification. PR IHC-negative (n=11); PR IHC-positive (n=12).

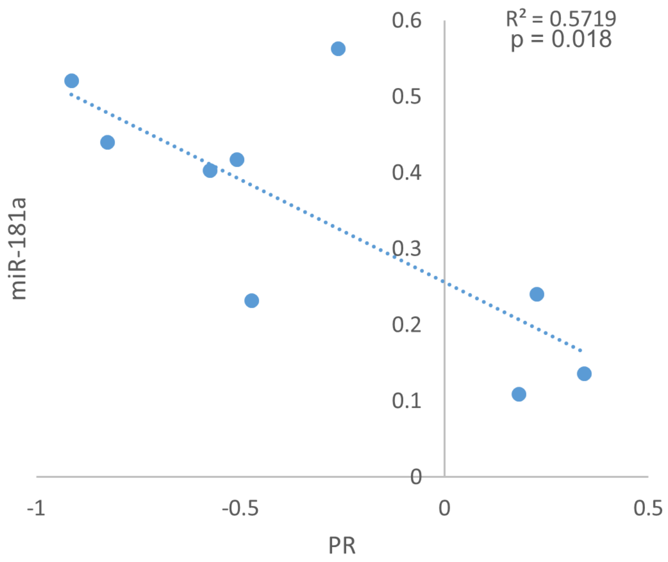

Supplementary Figure S2: Expression fold change of miR-181a and PR mRNA in each breast cancer tumor and its' adjacent normal tissue, in PR IHC-negative samples (n=9). Levels of miR-181a and PR mRNA were determined by quantitative RT-PCR analysis. The comparative threshold cycle (Ct) method was used for quantification. Values are presented in log scale. Line represents the linear regression analysis; R^2 (Goodness-of-fit) for the linear regression analysis and p-value are indicated.

Supplementary Table S1. PR mRNA, miR-181a, miR-23a and miR-26b expression Fold change (tumor sample vs. adjacent normal tissue)), according to PR mRNA expression fold change

	Patient no.	PR mRNA	miR-181a	miR-23a	miR-26b	PR-IHC status
PR down-regulation group	15	0.122	3.320	1.382	1.009	Negative
	16	0.149	2.751	1.085	5.066	Negative
	21	0.267	2.529	0.804	1.922	Negative
	5	0.310	2.614	2.767	5.113	Negative
	28	0.338	1.706	1.087	1.469	Negative
	4	0.549	3.655	3.876	5.552	Negative
	26	0.598	0.720	0.344	0.224	Positive
	23	0.608	0.758	0.817	1.046	Positive
No Change	20	0.807	1.738	1.597	1.091	Positive
PR up-regulation group	1	1.525	1.285	4.544	5.407	Negative
	17	1.634	6.433	2.102	6.066	Positive
	2	1.683	1.740	0.879	2.937	Negative
	3	2.201	1.366	2.614	3.037	Negative
	8	2.523	0.816	0.394	2.548	Positive
	11	6.597	0.585	0.232	1.741	Positive
	27	7.812	0.355	0.272	0.198	Positive
	22	11.538	2.069	1.397	2.433	Positive
	24	12.007	2.061	0.729	0.302	Positive
	9	12.245	0.529	0.193	1.819	Positive

	%
6/8 Negative	75
2/8 Positive	25

	%
3/10 Negative	30
7/10 Positive	70

PR, progesterone receptor; ☐<0.8 ; ☐>1.2

Supplementary Table S2. PR mRNA, miR-181a, miR-23a and miR-26b expression Fold change (tumor sample vs. adjacent normal tissue), according to PR IHC status

Patient no.	PR mRNA	miR-181a	miR-23a	miR-26b	PR-IHC status
26	0.598	0.720	0.344	0.224	Positive
23	0.608	0.758	0.817	1.046	Positive
20	0.807	1.738	1.597	1.091	Positive
17	1.634	6.433	2.102	6.066	Positive
8	2.523	0.816	0.394	2.548	Positive
11	6.597	0.585	0.232	1.741	Positive
27	7.812	0.355	0.272	0.198	Positive
22	11.538	2.069	1.397	2.433	Positive
24	12.007	2.061	0.729	0.302	Positive
9	12.245	0.529	0.193	1.819	Positive
15	0.122	3.320	1.382	1.009	Negative
16	0.149	2.751	1.085	5.066	Negative
21	0.267	2.529	0.804	1.922	Negative
5	0.310	2.614	2.767	5.113	Negative
28	0.338	1.706	1.087	1.469	Negative
4	0.549	3.655	3.876	5.552	Negative
1	1.525	1.285	4.544	5.407	Negative
2	1.683	1.740	0.879	2.937	Negative
3	2.201	1.366	2.614	3.037	Negative

PR, progesterone receptor; ☐<0.8 ; ☐>1.2
